# Interval Cytoreductive Surgery and Cisplatin- or Paclitaxel-Based HIPEC for Advanced Ovarian Cancer

**DOI:** 10.1001/jamanetworkopen.2025.17676

**Published:** 2025-06-26

**Authors:** Salud González Sánchez, Jorge García Fernández, Pedro Antonio Cascales-Campos, Alida Gonzalez Gil, Israel Manzanedo, Fernando Pereira Perez, Daniel Díaz Gómez, Carlos González-de Pedro, Enrique Asensio Diaz, David Pacheco Sanchez, Aranzazu Prada-Villaverde, Isabel Jaén Torrejimeno, Javier Lacueva, Iban Caravaca-Garcia, Juan Torres-Melero, Susana Sanchez-García, Eduardo Díaz Reques, César Ramírez Plaza, Alberto Gutiérrez-Calvo, Luis González Bayón, Rafael Morales-Soriano, Fernando López-Mozos, Lana Bjelic, Julio Galindo Álvarez, Manuel Emilio Marcello Fernandez, Estrella Turienzo Santos, Alberto Titos García, Rosa Álvarez Seoane, Manuel Artiles Armas, Emilio Terol Garaulet, Pedro Villarejo Campos, Enrique Boldó Roda, Cristina Rihuete Caro, Alfonso García Fadrique, Álvaro Arjona-Sánchez

**Affiliations:** 1 Unit of Surgical Oncology, Reina Sofia University Hospital, Cordoba, Spain; 2 GE09 Research in Peritoneal and Retroperitoneal Oncologic Surgery Group, Maimonides Biomedical Research Institute of Cordoba, Reina Sofía University Hospital, University of Cordoba, Córdoba, Spain; 3 Unit of Gynecology, Reina Sofia University Hospital, Cordoba, Spain; 4 Peritoneal Surface Malignancy Unit, Department of Surgery Clinic and University Hospital “Virgen de la Arrixaca,” University of Murcia, Murcia, Spain; 5 Peritoneal Carcinomatosis Unit, Department of General and Digestive Surgery, Fuenlabrada University Hospital, Madrid, Spain; 6 Unit of Surgical Oncology Peritoneal and Retroperitoneal, Virgen del Rocio University Hospital, Seville, Spain; 7 Advanced Oncologic Surgery Unit, Department of General and Digestive Surgery, Hospital Río Hortega, Valladolid, Spain; 8 Department of HBP and Liver Transplant Surgery, Badajoz University Hospital, Badajoz, Spain; 9 Oncological Abdominal and Pelvic Surgery Unit, Department of General Surgery, University General Hospital of Elche, Elche, Spain; 10 Department Pathology and Surgery, Miguel Hernández University, San Juan de Alicante, Spain; 11 Department of General and Digestive Surgery, Torrecardenas University Hospital, Almería, Spain; 12 Unit of Surgical Oncology, University Hospital Ciudad Real, Ciudad Real, Spain; 13 Unit of Surgery, Hospital San Chinarro, Madrid, Spain; 14 Unit of Surgical Oncology, Hospital Quiron, Malaga, Spain; 15 Unit of Surgical Oncology, University Hospital Principe de Asturias, Alcala Henares, Madrid, Spain; 16 Unit of Surgical Oncology, University Hospital Gregorio Marañon, Madrid, Spain; 17 Unit of Surgical Oncology, University Hospital Son Espases, Palma de Mallorca, Spain; 18 Unit of Surgical Oncology, University Hospital Clinic of Valencia, Valencia, Spain; 19 Unit of Surgical Oncology, Hospital Moises Broggi, Barcelona, Spain; 20 Hospital Universitario Ramón y Cajal, Insititute Ramón y Cajal de Investigación Sanitaria (IRYCIS) Ramon y Cajal; 21 University Hospital Alcorcon Foundation, Madrid, Spain; 22 Unit of Surgical Oncology, University Hospital Central Asturias, Asturias, Spain; 23 Unit of Surgery, University Hospital Regional Malaga, Malaga, Spain; 24 Unit of Surgical Oncology, University Hospital La Coruña, Spain; 25 Unit of Surgical Oncology, Hospital Negrin Las Palmas de Gran Canaria, Las Palmas, Spain; 26 Unit of Peritoneal Surgery, University General Reina Sofia Hospital, Murcia, Spain; 27 Unit of Surgical Oncology, University Hospital Fundacion Jimenez Diaz, Madrid, Spain; 28 Unit of Surgical Oncology, Hospital Provincial Castellon, Castelló, Spain; 29 Unit of Surgery, Infanta Elena University Hospital, Madrid, Spain; 30 Unit of Surgical Oncology, Oncologic Valencia Institute, Valencia, Spain

## Abstract

**Importance:**

Ovarian cancer, often diagnosed at advanced stages, presents significant challenges in treatment and survival. Evaluation of different hyperthermic intraperitoneal chemotherapy (HIPEC) regimens could provide crucial insights to improve patient outcomes.

**Objective:**

To evaluate whether HIPEC with paclitaxel (HIPEC-paclitaxel) is associated with similar oncological outcomes as HIPEC with cisplatin (HIPEC-cisplatin) in patients with advanced ovarian cancer undergoing interval cytoreductive surgery (iCRS).

**Design, Setting, and Participants:**

This multicenter retrospective cohort study included patients with advanced ovarian cancer who received iCRS and HIPEC. Patients with primary or secondary surgical procedures or nonovarian cancers were excluded. Data came from the National Registry of Peritoneal Carcinomatosis, which includes 27 Spanish specialized peritoneal oncology centers. Cases were included from January 2012 to December 2022. The study used propensity score matching to balance the groups and ensure comparability.

**Exposure:**

HIPEC-cisplatin and HIPEC-paclitaxel, administered during iCRS. The HIPEC regimen was selected based on the standard clinical protocol for advanced ovarian cancer.

**Main Outcomes and Measures:**

The primary end points were overall survival (OS) and disease-free survival (DFS). The secondary end point was the rate of complications in each group. These outcomes were predefined prior to data collection.

**Results:**

A total of 846 patients (mean [SD] age, 59.04 [11.01] years) were included (325 [38.4%] in HIPEC-cisplatin group; 521 [61.6%] in HIPEC-paclitaxel group), and 199 patients in each group were propensity score matched. Among these 398 matched patients, the HIPEC-paclitaxel group had similar DFS and OS compared with the HIPEC-cisplatin group. Additionally, similar morbidity was observed. Equivalence in OS and DFS was observed during the initial 20 and 15 months of follow-up, respectively, with an equivalence margin of 0.1 respectively.

**Conclusions and Relevance:**

In this cohort study of patients with advanced ovarian cancer, HIPEC-paclitaxel was associated with comparable oncologic outcomes as HIPEC-cisplatin, suggesting that it could be a viable alternative. These findings support its use, especially in patients in whom cisplatin could be contraindicated. Further studies may help refine treatment protocols and improve patient-specific outcomes.

## Key Points

**Question:**

Is paclitaxel-based hyperthermic intraperitoneal chemotherapy (HIPEC) safe and associated with equivalent outcomes as cisplatin-based HIPEC in the treatment of advanced ovarian cancer after interval cytoreductive surgery?

**Findings:**

In this cohort study with 846 patients, the use of paclitaxel-based HIPEC was associated with similar overall survival and disease-free survival as cisplatin-based HIPEC in both matched and unmatched cohorts. Paclitaxel-based HIPEC was not associated with increased morbidity.

**Meanings:**

These findings suggest that paclitaxel-based HIPEC could be an alternative to cisplatin-based HIPEC.

## Teaser

This cohort study examines whether outcomes among women in Spain with advanced ovarian cancer are different when they are treated with paclitaxel-based hyperthermic intraperitoneal chemotherapy (HIPEC) vs cisplatin-based HIPEC during interval cytoreductive surgery.

## Introduction

Ovarian cancer is the second leading cause of death among all gynecological cancers. The estimated number of new cases in the GLOBOCAN registry in 2022 was 324 398 cases, with 206 839 deaths.^1^ More than two-thirds of patients are diagnosed with advanced ovarian cancer (AOC). Ovarian cancer diagnosed in young women raises concerns about their fertility. If diagnosed during pregnancy, maternal and fetal factors must be considered. The most common and lethal tube-ovarian carcinoma is high-grade serous carcinoma (HGSC).^2^

The standard treatment for AOC is cytoreductive surgery with minimal or null residual disease and adjuvant chemotherapy based on carboplatin and paclitaxel.^3^ Neoadjuvant chemotherapy based on carboplatin and paclitaxel followed by interval cytoreductive surgery (iCRS) is considered a valid alternative to primary surgical treatment in patients with high burden HGSC, achieving similar survival outcomes with fewer perioperative complications.^4,5,6,7^

The use of additional therapies during surgery, such as hyperthermic intraperitoneal chemotherapy (HIPEC), is considered to have potential benefits in progression-free survival (PFS) and overall survival (OS) during iCRS,^8,9,10^ but it is controversial.^11^ HIPEC with iCRS has been tested in phase 3 clinical trials using cisplatin, with a dose of approximately 75 to 100 mg/m^2^ for 60 to 90 minutes^8,9,10^; however, paclitaxel-based HIPEC at 120 mg/m^2^ for 60 minutes has less evidence.^12,13^ Paclitaxel-based HIPEC is routinely used in multiple centers and also may be a substitute for cisplatin for patients with frailty, in the presence of kidney failure, or when a patient has any intolerance to platins.^14^

The aim of this study is to compare the early oncological outcomes in a matched cohort of patients who underwent iCRS with HIPEC based on cisplatin vs paclitaxel. For this purpose, a multicenter national registry was used. These results could indicate that paclitaxel-based HIPEC is a safe and effective alternative to cisplatin for older patients, patients with kidney failure, or patients who are intolerant to platins.

## Methods

### Study Design

This multicenter retrospective cohort study used the National Registry of Peritoneal Carcinomatosis (REGECOP), which includes 27 centers involved in the treatment of peritoneal carcinomatosis. The study and the use of the registry were approved by the ethics committee of the University Hospital Fuenlabrada and by each local ethics committee. Informed consent was not required because anonymized data were provided by REGECOP. This report follows the Strengthening the Reporting of Observational Studies in Epidemiology (STROBE) reporting guideline for cohort studies.

### Participants

**Figure 1.**
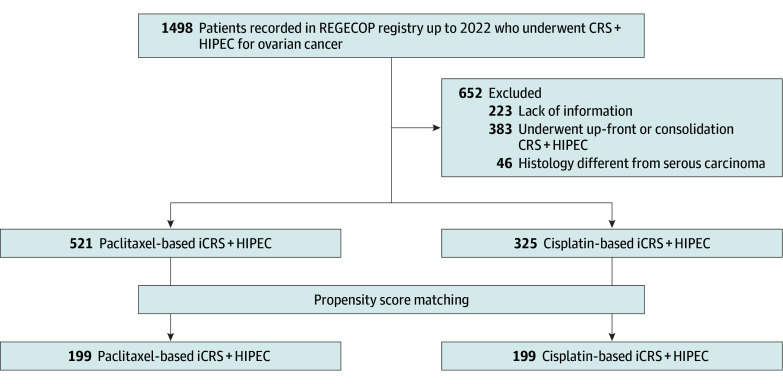
Study Flowchart CRS indicates cytoreductive surgery; HIPEC, hyperthermic intraperitoneal chemotherapy; iCRS + HIPEC, interval CRS with HIPEC; and REGECOP, National Registry of Peritoneal Carcinomatosis.

Patients included in REGECOP who underwent iCRS with cisplatin- or paclitaxel-based HIPEC for primary carcinomatosis of ovarian origin from 2002 to 2022 were included in the study (eTable in Supplement 1). Patients were diagnosed with ovarian carcinoma high grade stage IIIc or IV, per the International Federation of Gynecology and Obstetrics (FIGO). All the patients received neoadjuvant and adjuvant chemotherapy based on national protocols, including maintenance with bevacizumab since 2015, maintenance with poly (ADP-ribose) polymerase (PARP) inhibitors in *BRCA-*altered disease since 2019, and PARP with bevacizumab in *BRCA*-altered or homologous recombination deficiency–positive disease since 2020. It is assumed that treatment distribution was similar between groups according to national protocols. Patients were excluded if they did not meet the inclusion criteria, such as upfront cytoreductive surgery, secondary cytoreductive surgery with HIPEC, or cancer of a nonovarian origin; patients with missing information were also excluded (Figure 1).

### Procedures

Patients underwent iCRS with HIPEC in reference units after stabilization or partial response after 3 to 4 cycles of carbotaxol-based chemotherapy (IIIc FIGO), assessed by at least a computed tomography scan and cancer antigen 125 levels; in some cases, staging laparoscopy or positron emission tomography scans were performed to assess the resectability. iCRS with HIPEC was performed by laparotomy and after complete abdominal cavity exploration and peritoneal cancer index (PCI) evaluation to determinate resectability.^15^ Multivisceral resections, peritonectomy procedures, and total hysterectomy with bilateral adnexectomy were performed to achieve a complete cytoreduction according to the completeness of cytoreduction score.^16^ After completion of cytoreduction, HIPEC was administered using either paclitaxel or cisplatin as follows: (1) cisplatin dose was 75 to 100 mg/m^2^ in 4 L of dextrose-based peritoneal perfusion for 90 minutes at 42 to 43 °C; (2) paclitaxel was administered at 120 mg/m^2^ in 4 L of dextrose-based perfusion for 60 minutes at 42 to 43 °C. Open or closed HIPEC technique was recorded and evaluated. After surgery, patients received adjuvant chemotherapy according to the carbotaxol scheme. Morbidity was evaluated at 30 days using the Clavien-Dindo classification.

### Statistical Analysis

To determine the appropriate statistical tests, the Anderson-Darling test was used to assess the normality of the data and the Fligner-Killeen test was used to evaluate homoscedasticity. Based on these assessments, either the parametric *t* test or the nonparametric Mann-Whitney *U* test was selected for unmatched data. For matched data, paired *t* tests and paired Wilcoxon signed-rank tests were performed. When sample sizes were small in 1 or more categories, the Fisher exact test was chosen over the χ^2^ test. Additionally, the McNemar test was used to compare paired proportions in the matched samples.

Prior to propensity score matching, an exploratory analysis was conducted to identify important variables for matching. A logistic regression model was fitted to estimate the probability of being in the treatment group (ie, type of HIPEC drug) as a function of potential confounders. The treatment variable of interest was the use of paclitaxel or cisplatin. Initial variables considered were (1) age (in years), (2) PCI, (3) cytoreduction score, (4) whether the patient had undergone more than 4 previous procedures, and (5) open or closed surgical technique. To account for the imbalance in treatment group sizes, weights were adjusted according to the proportion of individuals in each treatment group. A 2-sided stepwise selection method was used to select significant variables for the final model.

Propensity scores (PSs) were estimated using the logistic regression model. The logit of the propensity scores (log[PS/(1 − PS)]) was used in matching instead of the PS because their distribution followed a normal distribution.^17,18^ In pairwise assignment (matched), the optimal method without resampling was used. The ratio was 1:1.

A diagnosis of the matching was carried out to check that it was performed correctly. The balance of the covariates was checked before and after matching. A threshold of 0.25 was set for the balance of covariates using the standardized mean difference (SMD). In our case, we used a threshold of 0.25 for the covariate balance and a threshold of 0.05 for the Kolmogorov-Smirnov (KS) test. In addition to the SMD, the variance ratio (VR) was used to check the balance of the covariates. A threshold of 2 was set for the VR. A love plot was performed to check the balance of the covariates before and after matching (SMD and KS statistics). Finally, a paired data test was performed to check the balance of covariates (1:1) (eFigure 1 in Supplement 1).

A logistic regression model was fitted to evaluate the occurrence of morbidity as a function of the treatment (cisplatin vs paclitaxel). The dependent variable was the binary outcome of morbidity (presence or absence of complications). The primary independent variable of interest was the type of treatment, with cisplatin as the reference group.

Survival distributions for both OS and disease-free survival (DFS) were estimated using the Kaplan-Meier method. Time at risk was considered from the surgical procedure to disease relapse or death (DFS) and to death for any cause (OS) in months; censored patients were considered patients at the end of follow-up without any event. Hazard ratios (HRs) for the treatment effect size, with cisplatin as the reference group, were estimated using the Cox proportional hazards regression model. To assess the proportional hazards assumption, we used log-log survival plots and the Grambsch and Therneau test for nonproportionality.

Furthermore, an equivalence test was performed to compare the survival curves of cisplatin and paclitaxel. Pointwise confidence intervals for the difference in survival probabilities were estimated using the delta method. The hypotheses for the equivalence test were defined as follows:

H_0_:∣S_cisplatin_−S_paclitaxel_∣≥ϵ

H_1_:∣ S_cisplatin_−S_paclitaxel_ ∣<ϵ

where S_cisplatin_ and S_paclitaxel_ represent the survival probabilities at time *t* for cisplatin and paclitaxel, respectively, and ϵ denotes the prespecified equivalence margin (set at 0.1). To visually assess the equivalence margin across the survival curves, mean differences and their corresponding 95% CIs (using the delta method) were plotted against time. All statistical analyses were conducted using R version 4.3.3 (R Project for Statistical Computing).

## Results

A total of 846 patients from the REGECOP national registry underwent iCRS with cisplatin-based HIPEC (n = 325) or with paclitaxel-based HIPEC (n = 521) from 27 reference centers (eTable in Supplement 1) belonging to the REGECOP (Figure 1). A hypothesis test was performed on the matched variables to test for significant differences between the 2 treatment groups (cisplatin- vs paclitaxel-based HIPEC). There were significant differences in age, HIPEC technique, PCI, having had more than 4 peritonectomy procedures, and cytoreduction completeness (Table 1).

**Table 1.** Nonmatched Demographic and Perioperative Characteristics

Variable	Patients, No. (%)			*P* value
	Cisplatin (n = 325)	Paclitaxel (n = 521)	Total (N = 846)	
Age, mean (SD), y	60.26 (11.02)	58.27 (10.95)	59.04 (11.01)	.006
HIPEC technique				
Closed	120 (36.92)	90 (17.27)	210 (24.82)	<.001
Open	205 (63.08)	431 (82.73)	636 (75.18)	
PCI score, mean (SD)	12.10 (8.47)	16.02 (9.21)	14.49 (9.12)	<.001
≥ 4 Peritonectomy procedures	111 (55.50)	290 (66.36)	401 (62.95)	.008
Completeness of cytoreduction				
0	306 (94.44)	454 (88.67)	760 (90.91)	.005
1-3	18 (5.56)	58 (11.33)	76 (9.09)	
Morbidity^a^				
Grade I	9 (4.39)	36 (15.52)	45 (10.30)	<.001
Grade II	167 (81.46)	68 (29.31)	235 (53.78)	
Grade IIIA	7 (3.41)	22 (9.48)	29 (6.64)	
Grade IIIB	9 (4.39)	32 (13.79)	41 (9.38)	
Grade IVa	9 (4.39)	60 (25.86)	69 (15.79)	
Grade IVb	3 (1.46)	7 (3.02)	10 (2.29)	
Grade V	1 (0.49)	7 (3.02)	8 (1.83)	

Abbreviations: HIPEC, hyperthermic intraperitoneal chemotherapy; PCI, peritoneal cancer index.

^a^ Data for 437 patients overall (205 in the cisplatin group and 232 in the paclitaxel group) reported. The Clavien-Dindo classification was used.

The model selected by the stepwise method was as follows:

log[*P*(HIPEC_Drug=Paclitaxel)/1 − *P*(HIPEC_Drug=Paclitaxel)] = α + β1(Age) + β2(PCI) + β3(HIPEC Open Technique).

Therefore, the final model for DFS included the following variables: age (odds ratio [OR], 0.99; 95% CI, 0.99-1.00; *P* = .03), PCI (OR, 1.04; 95% CI, 1.02-1.06; *P* < .001), and type of HIPEC technique (OR, 2.58; 95% CI, 1.83-3.67; *P* < .001).

### Sample Matching

According to the criteria established in the methods, we concluded that the variables were not balanced at the beginning and need to be adjusted. The SDM and VR of the variables were not acceptable, at greater than 0.25 and greater than 2 respectively; a 1:1 match was performed, We went from a sample size of 846 (521 + 325) to 398 (199 + 199) after matching. The balance of the covariates was significantly improved, with an SMD of 0.054 and VR of 0.91; the love plot is shown in eFigure 1 in Supplement 1). The final matched cohort was 1:1 with 199 patients in each group (Figure 1). Variables were balanced, as shown in Table 2.

**Table 2.** Matched Demographic and Perioperative Characteristics

Variable	Patients, No. (%)		*P* value
	Cisplatin (n = 199)	Paclitaxel (n = 199)	
Age, mean (SD), y	59.84 (11.26)	59.13 (10.95)	.51
HIPEC technique			
Closed	68 (53.12)	60 (46.88)	.09
Open	131 (48.52)	139 (51.48)	
PCI score, mean (SD)	13.05 (8.60)	12.55 (8.33)	.87
≥4 Peritonectomy procedures	111 (49.12)	115 (50.88)	.63
Completeness of cytoreduction			
0	187 (50.13)	186 (49.87)	.83
1-3	12 (48.00)	13 (52.00)	
Morbidity^a^			
Grade I	9 (10.97)	14 (17.07)	.52
Grade II	60 (73.17)	56 (68.29)	
Grade IIIA	0	0	
Grade IIIB	0	0	
Grade IVa	9 (10.97)	5 (6.09)	
Grade IVb	3 (3.65)	3 (3.65)	
Grade V	1 (1.21)	4 (4.88)	

Abbreviations: HIPEC, Hyperthermic intraperitoneal chemotherapy; PCI, peritoneal cancer index.

^a^ Data for 82 patients in each group reported. The Clavien-Dindo classification was used.

### Perioperative and Survival Outcomes

**Figure 2.**
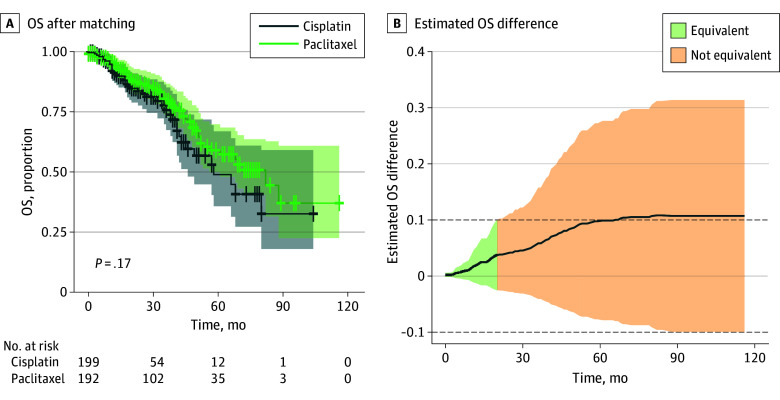
Overall Survival (OS) for Patients Treated With Cisplatin- and Paclitaxel-Based Hyperthermic Intraperitoneal Chemotherapy, After Matching A, Shaded areas indicate 95% CIs and crosses, censored cases. B, The shaded area represents the 95% CI using the delta method. The dashed horizontal line represents the equivalence margin of 10% difference in survival. Green areas indicate equivalence between the 2 treatments, while orange areas indicate nonequivalence.

**Figure 3.**
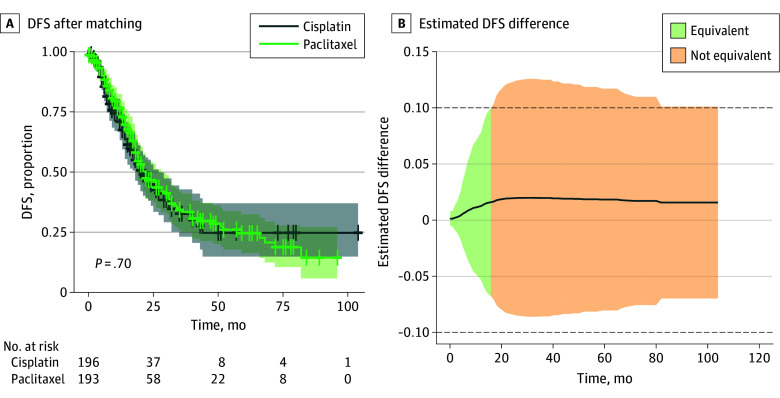
Disease-Free Survival (DFS) for Patients Treated With Cisplatin- and Paclitaxel-Based Hyperthermic Intraperitoneal Chemotherapy, After Matching A, Shaded areas indicate 95% CIs and crosses, censored cases. B, The shaded area represents the 95% CI using the delta method. The dashed horizontal line represents the equivalence margin of 10% difference in survival. Green areas indicate equivalence between the 2 treatments, while orange areas indicate nonequivalence.

After logistic regression in the matched population, paclitaxel-based HIPEC was not associated with an increase of morbidity with an OR of 1.32 (95% CI, 0.99-1.76; *P* = .06). Kaplan-Meier curves were fitted to evaluate OS and DFS as a function of treatment (cisplatin vs paclitaxel). Additionally, a Cox model was applied to determine the hazard ratio (HR) of the treatment in the matched population. In the matched model, the median OS for the cisplatin group was 58 (95% CI, 46-∞) months, and the median OS for the paclitaxel group was 82 (95% CI, 56-∞) months. Assuming cisplatin as the reference group, the HR was 0.74 (95% CI, 0.49-1.13; *P* = .16), indicating no significant difference between the treatment groups (Figure 2A; eFigure 3 in Supplement 1). Equivalence in OS was observed during the initial 20 months of follow-up with an equivalence margin (ϵ) of 0.1. After that, paclitaxel did not appear to be inferior to cisplatin, with a noninferiority margin of 0.1 (Figure 2B). The median DFS for the cisplatin group was 20 (95% CI, 18-27) months. The median DFS for the paclitaxel group was 21 (95% CI, 18-28) months, with an HR of 0.95 (95% CI, 0.72-1.25; *P* = .70, indicating no significant difference between the treatment groups (Figure 3A; eFigure 3 in Supplement 1). Equivalence in DFS was observed during the initial 15 months of follow-up with an equivalence margin (ϵ) of 0.1 (Figure 3B). Unmatched population survival curves appear in eFigure 2 in Supplement 1; no differences were observed.

## Discussion

iCRS combined with HIPEC remains controversial. The use of HIPEC in ovarian carcinomatosis has been supported by the publication of phase 3 clinical trials showing survival benefits with its use.^8,9,10^ All of these trials used cisplatin as the main drug for HIPEC, but paclitaxel is used by many groups for HIPEC indications in daily clinical practice.^12,13^ The present study found that the use of HIPEC with paclitaxel was associated with similar outcomes as cisplatin, and thus, it may be a valuable alternative for patients who are intolerant or resistant to platins or for patients with frailty or with deteriorating kidney function.

iCRS in advanced ovarian carcinoma has shown similar oncological outcomes with better perioperative results, improved rate of complete cytoreduction, fewer stomas, and better recovery.^4,6^ Perhaps these advantages do not impact the survival results, but certainly they improve the quality of life for patients with advanced ovarian cancer.^4,5,6^ iCRS is the most widespread practice for advanced ovarian carcinoma with high tumor burden and high-grade serous histology because it results in better postoperative outcomes^3^; this is the scenario in which our study took place. Our study selected this specific group of patients to evaluate the use of different drugs for iCRS and HIPEC, excluding other types of patients for whom iCRS is not indicated.^3^

HIPEC allows for locoregional administration of high doses of cytostatic agents in a single session, avoiding systemic adverse events and repeat sessions that can have a low rate of adherence, as shown by Amstrong et al.^19^ The use of HIPEC is controversial today; however, survival benefits have been demonstrated when used in the context of iCRS, improving PFS and OS.^8,9,10,19^ In the OVHIPEC-1 trial, patients in the experimental arm received iCRS with HIPEC using cisplatin (100 mg/m^2^ for 90 minutes) compared with the control group who received iCRS. The primary end point was PFS, and the study found a sustained survival benefit in the long-term analysis.^20^ Less strong evidence came from other phase 3 trials.^9,10^ A Korean study showed similar positive results only in the context of iCRS but not in primary CRS.^9^ In this trial, the survival benefit was obtained in a stratified analysis for the iCRS group, but the global results did not show a benefit with the use of HIPEC with cisplatin (75 mg/m^2^ for 90 minutes). The last trial^10^ was closed before completion of recruitment and enrolled an unpowered population, but the results showed a survival benefit with the use of HIPEC for iCRS using a low dose of cisplatin (75 mg/m^2^ for 60 minutes). The later evidence constitutes the strongest evidence to recommend HIPEC for iCRS in advanced ovarian cancer. Although the oncological surgeon community considers this sufficient to recommend HIPEC with iCRS, medical oncologists and gynecologic oncologists have presented counter positions.^3,21,22^

The use of paclitaxel in HIPEC is not common mainly for historical reasons, such as paclitaxel not having thermal synergism to enhance its toxic effects and it being a cell-cycle dependent drug.^23^ The use of hyperthermia is justified because it has a cytotoxic effect per se.^23^ One study analyzed this issue,^24^ and although no survival differences were observed when intraperitoneal paclitaxel was administered in hyperthermia vs normothermia, for both groups, paclitaxel showed adequate pharmacokinetics with reduction of cell cycle and proliferation markers in the hyperthermia group. In a recent collaborative publication on the use of HIPEC in advanced ovarian cancer,^25^ approximately 10% of the cases were treated with paclitaxel instead of cisplatin. In the present study, the use of paclitaxel was more common, with 521 of 846 patients (61.6%) receiving paclitaxel. This frequent use could be related to the safe profile that intraperitoneal paclitaxel presents compared with cisplatin, avoiding the need for tyosulfate to protect kidney function.^8^ Another advantage of HIPEC with paclitaxel is less systemic absorption compared with cisplatin due to paclitaxel’s high molecular weight (853.9 g/mol).^14^ Some studies have focused on the use of paclitaxel for HIPEC with excellent results, such as the C-HOC trial,^23^ which showed a benefit in tumor response with promising results for PFS; the HIPECOVA trial,^13^ which highlighted the potential benefit of HIPEC-associated cytoreduction with paclitaxel, particularly in selected patients with ovarian cancer and lower Peritoneal Surface Disease Severity Score indices; and a comparative study^26^ that showed similar results using paclitaxel or cisplatin during HIPEC. To our knowledge, our study is the first comparative matched study in a large population to observe the same outcomes with cisplatin and paclitaxel, making the latter a valuable alternative to cisplatin with the same major morbidity and no differences in DFS.

### Limitations

This study has limitations due to its inherent retrospective nature, but we only selected cases with complete information, excluding cases with missing information. *BRCA* alteration status was not recorded in the national registry; however, maintenance therapy was administered according to national protocols being balanced between groups. Propensity score matching was used to balance the 2 populations into 2 comparable groups with no statistical differences in the demographic and perioperative characteristics, and an accuracy test was used to evaluate the matched population. After the matching procedure, the resulting sample size was moderate, which could create the possibility of residual confounding using strict equivalence margins. The chosen end point was DFS given that our aim was to evaluate the outcomes of this locoregional therapy and not to use OS as an end point because multiple therapies could be used for relapses, which makes it so difficult to form strong conclusions about the outcomes associated with HIPEC.

## Conclusions

Our study suggests that cisplatin and paclitaxel are 2 safe and effective drugs to be used for HIPEC in iCRS for advanced ovarian cancer. As cisplatin is the preferred drug according to strong evidence, paclitaxel could be a valuable alternative for patients with any contraindication to cisplatin, with similar oncological and perioperative outcomes.

## Notes

Supplement 1. **eTable.** Patients Collected Per Center and Cisplatin-HIPEC Rate per Center **eFigure 1.** Love Plot Showing Standardized Mean Differences and Variance Ratios Before and After Matching **eFigure 2.** Survival Curves for the Unmatched Data **eFigure 3.** Log-Log Plot Showing the Complementary Log-Log Transformation of the Survival Curves for the Matched Data for Cisplatin and Paclitaxel

Supplement 2.
Nonauthor Collaborators

Supplement 3.
Data Sharing Statement
