# Supplementary material for: Interval Cytoreductive Surgery and Cisplatin- or Paclitaxel-Based HIPEC for Advanced Ovarian Cancer
Source: JAMA Netw Open. 2025 Jun 26;8(6):e2517676. doi: 10.1001/jamanetworkopen.2025.17676 (PMC12203279; doi:10.1001/jamanetworkopen.2025.17676)
Supplement: Supplement 1. — eTable. Patients Collected Per Center and Cisplatin-HIPEC Rate per Center eFigure 1. Love Plot Showing Standardized Mean Differences and Variance Ratios Before and After Matching eFigure 2. Survival Curves for the Unmatched Data eFigure 3. Log-Log Plot Showing the Complementary Log-Log Transformation of the Survival Curves for the Matched Data for Cisplatin and Paclitaxel [file jamanetwopen-e2517676-s001.pdf]

Supplemental Online Content

González Sánchez S, García Fernández J, Cascales-Campos PA, et al; REGECOP collaborators. Interval cytoreductive surgery and cisplatin- or paclitaxel-based HIPEC for advanced ovarian cancer. *JAMA Netw Open.* 2025;8(6):e2517676. doi:10.1001/jamanetworkopen.2025.17676

eTable. Patients Collected Per Center and Cisplatin-HIPEC Rate per Center

eFigure 1. Love Plot Showing Standardized Mean Differences and Variance Ratios Before and After Matching

eFigure 2. Survival Curves for the Unmatched Data

eFigure 3. Log-Log Plot Showing the Complementary Log-Log Transformation of the Survival Curves for the Matched Data for Cisplatin and Paclitaxel

This supplemental material has been provided by the authors to give readers additional information about their work.

© 2025 González Sánchez S et al. *JAMA Network Open*.

eTable. Patients Collected Per Center and Cisplatin-HIPEC Rate per Center

**Center**	**Patients**	**Cisplatin rate %**
Dr. Negrín	9	0.60%
Broggi	19	1.27%
Infanta Elena	4	0.27%
HUUR	147	9.81%
Carlos Haya	12	0.80%
Jimenez Diaz	6	0.40%
Fundacion Alarcon	17	1.13%
Clinico Valencia	23	1.54%
Río Hortega	123	8.21%
Principe Asturias	30	2.00%
Sanchinarro	43	2.87%
Ramon y Cajal	17	1.13%
Torrecárdenas	54	3.61%
Gregorio Marañón	27	1.80%
Son Espases	25	1.67%
Elche	87	5.81%
HUCA	12	0.80%
Quirón Málaga	35	2.34%
Castellón	4	0.27%
A Coruña	11	0.73%
HURS Murcia	6	0.40%
HU Badajoz	122	8.14%
Instituto Valenciano de Oncología	1	0.07%
Fuenlabrada	218	14.55%
Arrixaca	258	17.22%
Ciudad Real	45	3.01%
HURS Córdoba	143	9.55%
**TOTAL**	1498	100%

© 2025 González Sánchez S et al. *JAMA Network Open*.

eFigure 1. Love Plot Showing Standardized Mean Differences (SMD, Figure A) and Variance Ratios (VR, Figure B) Before (Red Circle) and After Matching (Blue Triangle)

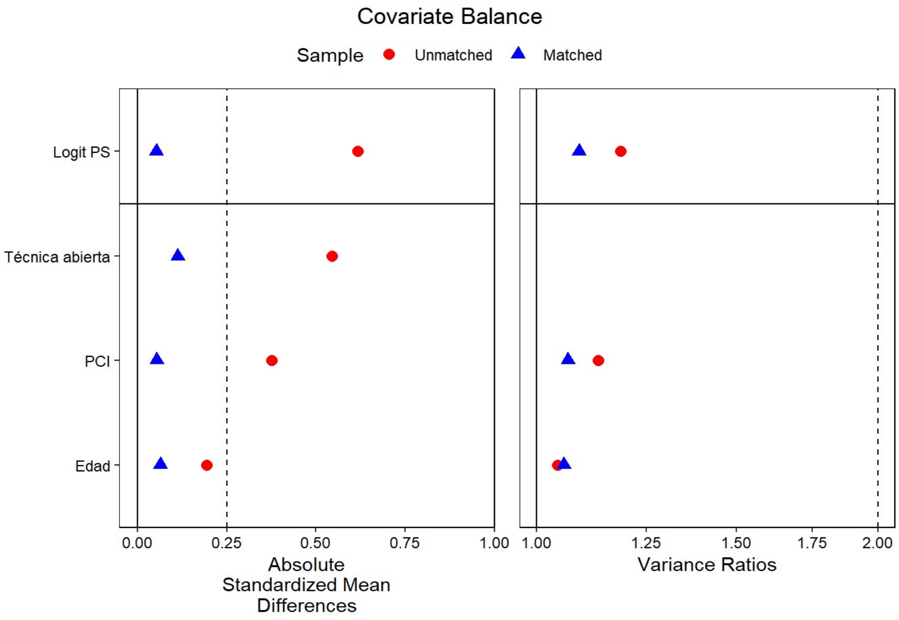

	SMD	SMD.Threshold	VR	VR.Threshold	KS
Logit PS	0.054	Balanced, <0.25	0.917	Balanced, <2	0.06
Age	-0.064	Balanced, <0.25	0.946	Balanced, <2	0.095
PCI	-0.054	Balanced, <0.25	0.938	Balanced, <2	0.045
Open tech	0.04	Balanced, <0.25			0.04

eFigure 1. Love Plot Showing Standardized Mean Differences (SMD, figure A) and Variance Ratios (VR, figure B) Before (red circle) and After Matching (blue triangle)

Vertical dashed line represents the threshold of unbalance (SMD > 0.25 and VR > 2). PCI = Peritoneal Cancer Index, Age = Age at inclusion, Open tech (Ref: Closed) = Open technique (Reference: Closed) and Logit PS = Logit of the propensity score.

© 2025 González Sánchez S et al. *JAMA Network Open*.

eFigure 2. Survival Curves for the Unmatched Data

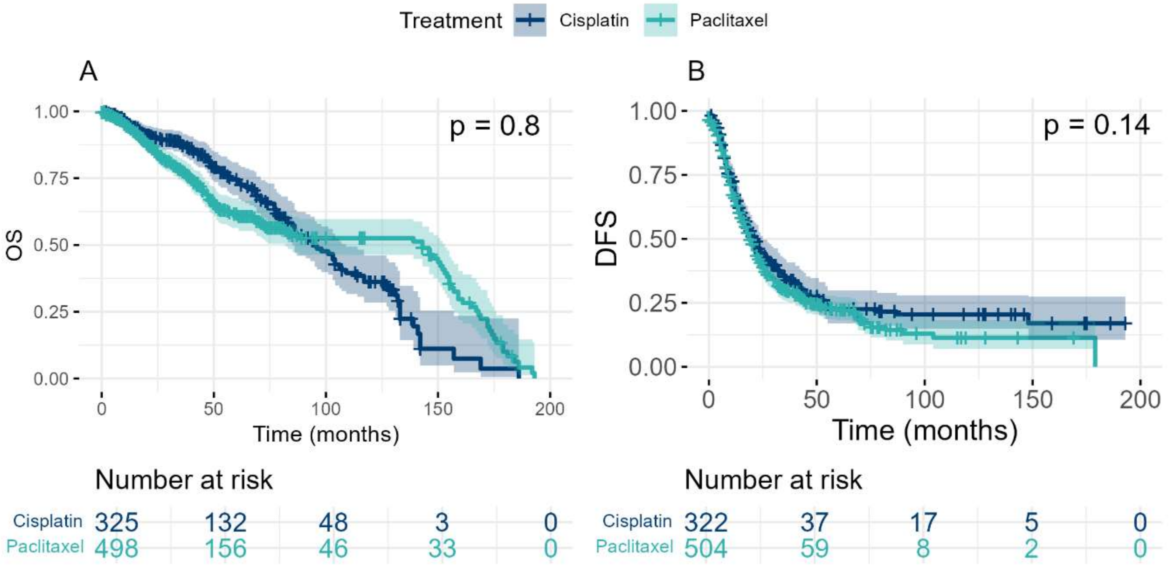

A: Overall survival (OS) and B: Disease-free survival (DFS) for Cisplatin and Paclitaxel. The tables show the number of patients at risk at different time points. The log-rank test p-value is shown in the upper right corner of each plot.

© 2025 González Sánchez S et al. *JAMA Network Open*.

eFigure 3. Log-Log Plot Showing the Complementary Log-Log Transformation of the Survival Curves for the Matched Data for Cisplatin and Paclitaxel

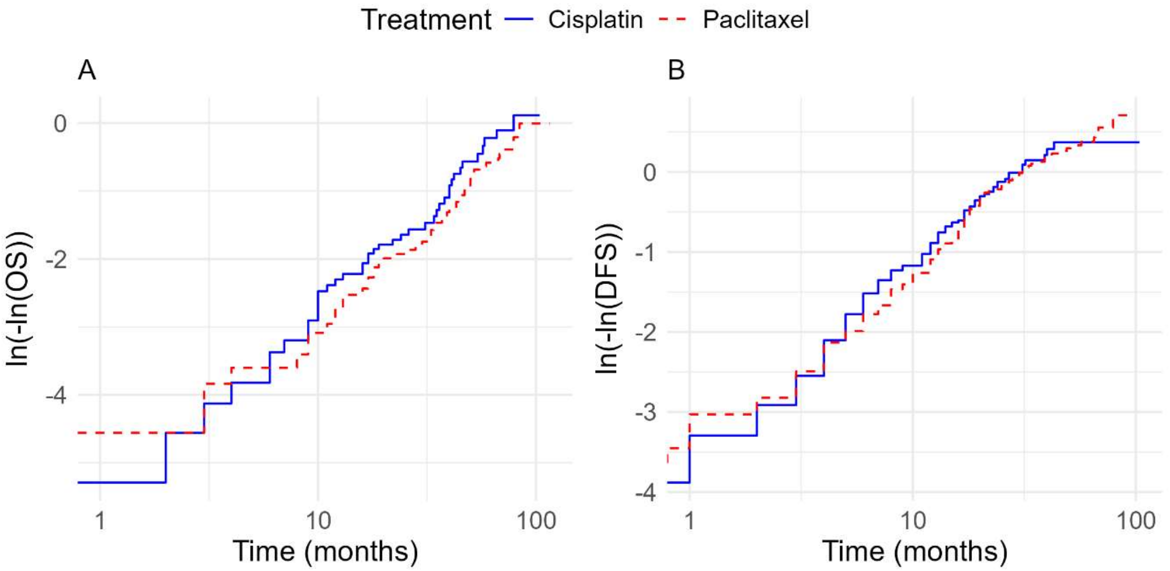

A: Overall survival, B: Disease-free survival.

© 2025 González Sánchez S et al. *JAMA Network Open*.
