# Supplementary material for: Interval Cytoreductive Surgery and Cisplatin- or Paclitaxel-Based HIPEC for Advanced Ovarian Cancer
Source: JAMA Netw Open. 2025 Jun 26;8(6):e2517676. doi: 10.1001/jamanetworkopen.2025.17676 (PMC12203279; doi:10.1001/jamanetworkopen.2025.17676)
Supplement: Supplement 2. — Nonauthor Collaborators [file jamanetwopen-e2517676-s002.pdf]

Supplemental Online Content: Nonauthor Collaborators
*First name, last name, and suffix (if applicable) are required and will appear in PubMed.

*Group Name(s): REGECOP National Registry							
***First Name and Middle Initial(s)**	***Last Name**	***Suffix (eg, Jr, III)**	Academic Degrees	Institution	Location (city, state/province, country)	Role or Contribution, eg, chair, principal investigator	Group (if more than 1 Group listed in the byline) and/or Subgroup (eg, Steering Committee)
Pablo	Lozano-Lominchar		MD	Peritoneal Carcinomatc	Madrid	Collaborator	
Wenceslao	Vasquez		MD	Peritoneal Carcinomatc	Madrid	Collaborator	
Elena	Gil-Gomez		MD	Departamento de Cirug	Murcia	Collaborator	
Melissa	Granados		MD	Unit of Surgical Oncology	Cordoba	Collaborator	
Mari Carmen	Vazquez-Borrego		PhD	Unit of Surgical Oncology	Cordoba	Collaborator	
Angela	Casado-Adam		phD	Unit of Surgical Oncology	Cordoba	Collaborator	
Lidia	Rodriguez Ortíz		MD	Unit of Surgical Oncology	Cordoba	Collaborator	
Blanca	Rufian		MD	Unit of Surgical Oncology	Cordoba	Collaborator	
Elena	Gonzalez			University Hospital Reina Sofia	Cordoba		
