# Supplementary material for: Interval Cytoreductive Surgery and Cisplatin- or Paclitaxel-Based HIPEC for Advanced Ovarian Cancer
Source: JAMA Netw Open. 2025 Jun 26;8(6):e2517676. doi: 10.1001/jamanetworkopen.2025.17676 (PMC12203279; doi:10.1001/jamanetworkopen.2025.17676)
Supplement: Supplement 3. — Data Sharing Statement [file jamanetwopen-e2517676-s003.pdf]

Data Sharing Statement

González Sánchez. Interval Cytoreductive Surgery and Cisplatin- or Paclitaxel-Based HIPEC for Advanced Ovarian Cancer. *JAMA Netw Open*. Published June 26, 2025.
doi:10.1001/jamanetworkopen.2025.17676

Data
Data available: Yes
Data types: Deidentified participant data
How to access data: Data will be sent after receiving a request to corresponding author for scientific purposes.
When available: With publication

Supporting Documents
Document types: Statistical/analytic code
How to access documents: Will be provided after contacting with corresponding author
When available: With publication

Additional Information
Who can access the data: anyone requesting the data, researchers whose proposed use of the data has been approved
Types of analyses: any
Mechanisms of data availability: contacting with corresponding author
